# Supplementary material for: Hybrid Models and Biological Model Reduction with PyDSTool
Source: PLoS Comput Biol. 2012 Aug 9;8(8):e1002628. doi: 10.1371/journal.pcbi.1002628 (PMC3415397; doi:10.1371/journal.pcbi.1002628)
Supplement: Text S4 — Complete source code for the PyDSTool package (version 0.88.120504). Includes API documentation and help files linking to web pages. This file is identical to the current public release on Sourceforge.net. (ZIP) [file pcbi.1002628.s004.zip › PyDSTool/html/PyDSTool.MProject.feature-class.html]

xml version="1.0" encoding="ascii"?


PyDSTool.MProject.feature


| Home | Trees | Indices | Help | PyDSTool |
| --- | --- | --- | --- | --- |

Package PyDSTool ::
Module MProject ::
Class feature

[hide private]

[frames] | no frames]

# Class feature

source code

```
object --+
         |
        feature
```

Known Subclasses:
:   - feature\_leaf
    - , feature\_node

---

End users of concrete sub-classes provide (required) evaluate method
and (optional) prepare, finish methods.


|  |  |
| --- | --- |
| Instance Methods | [hide private] |

|  |  |
| --- | --- |
| \_\_init\_\_(self, name, description=`'``'`, pars=None, ref\_traj=None)  x.\_\_init\_\_(...) initializes x; see x.\_\_class\_\_.\_\_doc\_\_ for signature | source code |

|  |  |
| --- | --- |
| \_\_hash\_\_(self)  hash(x) | source code |

|  |  |
| --- | --- |
| \_\_eq\_\_(self, other) | source code |

|  |  |
| --- | --- |
| \_\_ne\_\_(self, other) | source code |

|  |  |
| --- | --- |
| \_find\_idx(self)  Internal function for finding index in trajectory meshpoints at which containment first failed. | source code |

|  |  |
| --- | --- |
| \_\_call\_\_(self, target) | source code |

|  |  |
| --- | --- |
| set\_ref\_traj(self, ref\_traj)  May or may not be used by the feature. | source code |

|  |  |
| --- | --- |
| evaluate(self, target) | source code |

|  |  |
| --- | --- |
| prepare(self, target)  Operations to prepare for testing (optional). | source code |

|  |  |
| --- | --- |
| finish(self, target)  Operations to complete only if evaluate was True (optional). | source code |

|  |  |
| --- | --- |
| info(self) | source code |

|  |  |
| --- | --- |
| postprocess\_ref\_traj(self)  User-definable by overriding in a sub-class | source code |

|  |  |
| --- | --- |
| validate(self)  NOT YET IMPLEMENTED. | source code |

|  |  |
| --- | --- |
| reset\_metric(self) | source code |

**Inherited from `object`**:
`__delattr__`,
`__getattribute__`,
`__new__`,
`__reduce__`,
`__reduce_ex__`,
`__repr__`,
`__setattr__`,
`__str__`


|  |  |
| --- | --- |
| Properties | [hide private] |

**Inherited from `object`**:
`__class__`


|  |  |
| --- | --- |
| Method Details | [hide private] |

|  |  |
| --- | --- |
| \_\_init\_\_(self, name, description=`'``'`, pars=None, ref\_traj=None)  *(Constructor)* | source code |

x.\_\_init\_\_(...) initializes x; see x.\_\_class\_\_.\_\_doc\_\_ for
signature

Overrides: object.\_\_init\_\_: *(inherited documentation)*

|  |  |
| --- | --- |
| \_\_hash\_\_(self)  *(Hashing function)* | source code |

hash(x)

Overrides: object.\_\_hash\_\_: *(inherited documentation)*

|  |  |
| --- | --- |
| \_find\_idx(self) | source code |

Internal function for finding index in trajectory meshpoints at which
containment first failed. Defaults to returning None and must be
overridden by a class that has access to a mesh.

|  |  |
| --- | --- |
| set\_ref\_traj(self, ref\_traj) | source code |

May or may not be used by the feature. If not used, it will be
ignored.

|  |  |
| --- | --- |
| prepare(self, target) | source code |

Operations to prepare for testing (optional). Override in concrete
sub-class if desired

|  |  |
| --- | --- |
| finish(self, target) | source code |

Operations to complete only if evaluate was True (optional). Override
in concrete sub-class if desired

|  |  |
| --- | --- |
| validate(self) | source code |

NOT YET IMPLEMENTED. Would test for reachability of a feature? This is
not easy and may be unnecessary!

  


| Home | Trees | Indices | Help | PyDSTool |
| --- | --- | --- | --- | --- |

|  |  |
| --- | --- |
| Generated by Epydoc 3.0.1 on Fri May 4 15:24:06 2012 | http://epydoc.sourceforge.net |
